# The Role of Quantitative Indocyanine Green Angiography with Relative Perfusion Ratio in the Assessment of Gastric Conduit Perfusion in Oesophagectomy: A Retrospective Study

**DOI:** 10.3390/jcm15010184

**Published:** 2025-12-26

**Authors:** Lee Shyang Kyang, Nurojan Vivekanandamoorthy, Simeng Li, David Goltsman, Aldenb Lorenzo, Neil Merrett

**Affiliations:** 1 Upper Gastrointestinal Surgery Unit, Department of Surgery, Bankstown-Lidcombe Hospital, Bankstown, NSW 2200, Australia; nurojan.vivekanandamoorthy@health.nsw.gov.au (N.V.); simeng.li1@health.nsw.gov.au (S.L.); david.goltsman@health.nsw.gov.au (D.G.); aldenb.lorenzo@health.nsw.gov.au (A.L.); neil.merrett@health.nsw.gov.au (N.M.); 2 School of Medicine, The University of New South Wales Australia, Kensington, NSW 2052, Australia; 3 School of Medicine, Western Sydney University, Penrith, NSW 2751, Australia

**Keywords:** indocyanine green, icg, anastomotic leak, oesophageal cancer, oesophagectomy

## Abstract

**Background:** Anastomotic leak (AL) after esophagectomy remains a devastating complication. Indocyanine green (ICG) fluorescence angiography may mitigate this risk by enabling perfusion-guided anastomotic site selection. This study evaluates the feasibility of quantitative ICG angiography using the SPY-PHI QP^®^ system (Stryker AB, Malmö, Sweden) during gastric conduit reconstruction. **Methods:** Six patients undergoing esophagectomy (Ivor Lewis/McKeown) after neoadjuvant therapy were retrospectively identified. ICG angiography was performed intraoperatively, with perfusion at the gastric conduit quantified as a relative perfusion ratio (RPR) using the first duodenal segment as the reference (100%). Anastomotic sites were selected based on maximal RPR (threshold > 80%). Postoperative outcomes included AL incidence (radiological/clinical), complications (Clavien–Dindo), and 90-day mortality. **Results:** All patients (median age: 69 years) underwent successful perfusion assessment. Adenocarcinoma predominated (50%, 3/6), with most tumours at the gastroesophageal junction (Siewert II: 66%). Intraoperative RPR at anastomotic sites ranged from 80% to 100%. No anastomotic leaks occurred. Complications included Clavien–Dindo grade II (n = 3; respiratory infections) and grade IV (n = 2; reintubation). There was no 90-day mortality. **Conclusions:** Quantitative ICG angiography using the SPY-PHI QP^®^ system facilitated perfusion-guided anastomosis with no leaks observed. Standardising perfusion assessment based on an RPR threshold of >80% may enhance surgical safety, though larger studies are needed to validate these findings.

## 1. Introduction

Oesophageal cancer is one of the leading causes of cancer-related death worldwide [1]. Despite advancements in surgical techniques and perioperative care, oesophagectomy remains the cornerstone of curative treatment for patients seeking long-term survival. However, the procedure is associated with significant morbidity [2,3] and mortality [4,5], making it one of the most challenging gastrointestinal surgeries.

Among its complications, anastomotic leak (AL) is one of the most feared and life-threatening outcomes, with a reported incidence of approximately 10% [6]. Its consequences are severe, including an increased need for reintervention, prolonged hospitalisation, delayed nutritional initiation, elevated 90-day mortality, and poorer long-term oncological outcomes [7,8,9]. A critical risk factor for AL is inadequate blood supply to the tip of the gastric conduit [10,11]. The complexity of oesophageal reconstruction plays a key role in this risk. Typically, the conduit is fashioned using a tubularised gastric graft, perfused primarily by the right gastroepiploic arcade. However, perfusion at the proximal portion of the graft—where the anastomosis is created—is often tenuous, as the arcade rarely extends to the tip of the graft [12]. This vascular limitation increases the risk of ischemia at the anastomotic site, contributing to poor healing and potential leakage.

ICG is a tricarbocyanine fluorescent dye that, when injected intravenously, binds to plasma proteins and circulates within the vasculature. When exposed to near-infrared (NIR) light, it emits a fluorescent signal, allowing for dynamic visualisation of blood flow to the gastric conduit [13]. Multiple studies have demonstrated the utility of ICG fluorescence angiography in improving intraoperative decision-making [14,15]. ICG has been shown to aid in the selection of the optimal anastomotic site [12], reduce the likelihood of anastomotic ischemia [16], and, in some studies, contribute to a lower incidence of AL [17]. While evidence remains mixed regarding its direct impact on lowering AL rates [18,19,20], the integration of ICG into standard esophagectomy protocols may potentially guide surgical modifications to improve patient outcomes [21].

Quantitative ICG analysis remains a novel technology, and no method has yet been established as the gold standard [22]. One of the latest systems, SPY-PHI QP^®^ (Stryker AB, Malmö, Sweden), integrates the SPY Portable Handheld Imaging (SPY-PHI) system with advanced built-in software that quantifies relative perfusion, representing a promising step toward objective, reproducible perfusion assessment [23]. This study aimed to report our experience in using the SPY-Phi system in intraoperative decision-making during oesophagogastric reconstruction, assessing its impact on anastomotic integrity and postoperative complications.

## 2. Materials and Methods

### 2.1. Study Population

This is a single-surgeon institutional experience at a tertiary oesophagogastric referral centre across a one-year period. Retrospective data collection was conducted. Six patients who underwent oesophageal cancer resection with gastric conduit reconstruction following neoadjuvant therapy were identified. All patients underwent a preoperative workup, including upper gastrointestinal endoscopy, biopsy, and contrast-enhanced CT scans of the chest, abdomen, and pelvis.

### 2.2. Surgical Techniques

Procedures were performed by an upper gastrointestinal surgeon at the institution. The surgical approach was determined by tumour location, with patients undergoing one of the following oesophagectomy techniques: Ivor Lewis oesophagectomy [24] (open/hybrid) or McKeown oesophagectomy [25].

#### 2.2.1. Ivor Lewis Oesophagectomy

For Ivor Lewis oesophagectomy [24], the procedure was performed via a two-phase totally open or hybrid approach under general anaesthesia (double-lumen endotracheal tube). The totally open approach was selected for bulky tumours or when pneumoperitoneum was contraindicated (e.g., due to patient-related factors). In the hybrid surgery, the abdominal phase was performed laparoscopically in a supine position with three 10 mm and one 5 mm ports. Dissection began by mobilisation of the stomach along the gastrocolic ligament and ligation of short gastric vessels while preserving the right gastroepiploic arcade. The left gastric pedicle was dissected of nodal tissue and divided at the origin. This was followed by mediastinal dissection of the oesophagus. A wide gastric conduit was formed using an endoscopic stapler from the lesser curvature watershed area to the fundus.

The patient was then turned to the left decubitus position, and a right posterolateral thoracotomy incision was made at the fifth intercostal space. The pleura was incised, and the azygous vein was ligated. The oesophagus was mobilised “top-down” and transected proximally using a contour stapler. At this point, SPY ICG angiography was performed to assess the perfusion adequacy. The gastric conduit was then transposed into the right chest via the posterior mediastinal route, and an oesophagogastric anastomosis was created using a 25 mm circular stapler (OrVil™, Covidien, Mansfield, MA, USA). A feeding jejunostomy was placed, when necessary, depending on the patient’s nutritional state. Bilateral subcostal incision was used in the setting of an open abdominal approach.

#### 2.2.2. McKeown Oesophagectomy

The McKeown oesophagectomy [25] commenced with standardised abdominal and thoracic phases, as previously outlined. For cervical mobilisation, a longitudinal incision was made anterior to the sternocleidomastoid muscle, extending from 2 fingerbreadths above the cricoid cartilage to the suprasternal notch. Critical vessels—including the anterior facial vein, middle thyroid vein, and inferior thyroid artery—were ligated to optimise exposure. The strap muscles and carotid sheath were retracted laterally, revealing the cervical oesophagus. Meticulous dissection preserved the left recurrent laryngeal nerve. The proximal oesophagus was mobilised, transected, and subsequently anastomosed to the gastric conduit using the aforementioned technique.

### 2.3. Fluorescence Imaging with Indocyanine Green

Indocyanine green (ICG) angiography was administered intravenously at a dose of 5 mg (2 mL following dilution of a 25 mg vial of ICG with 10 mL of saline) via a central line. Perfusion assessment was performed using the SPY PHI^®^ system, which employs near-infrared light to detect ICG fluorescence, enabling real-time visualisation of tissue perfusion and vascular integrity assessment of the neo-oesophagus.

The SPY PHI^®^ clinical imaging system illuminates tissues with excitation wavelengths, allowing its sensor to capture and reproduce fluorescence images on a monitor. With the integration of quantification software, the SPY Phi^®^ (https://www.stryker.com/us/en/endoscopy/products/spy-phi.html; accessed on 22 May 2025) (Stryker, Amsterdam, The Netherlands) system provides objective perfusion data, generating a relative perfusion ratio (RPR) that quantifies blood flow to the neo-oesophagus.

**Figure 1:**
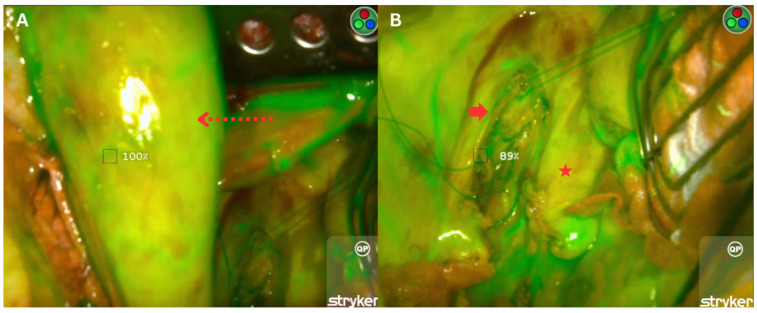
Intraoperative images showing (**A**) the relative perfusion ratio of the gastric conduit (red dotted arrow) and (**B**) the native perfusion ratio of the oesophageal stump (red arrow), located adjacent to the thoracic aorta (red star), during the thoracic phase of oesophagectomy.

To standardise perfusion measurements, the first part of the duodenum (D1) was designated as the 100% point of reference (POR) for perfusion and blood flow, against which all other measured points were compared. Perfusion was video-recorded for 180 s after fluorescence was first detected in D1, ensuring comprehensive vascular flow assessment. The software automatically identifies the frame corresponding to peak fluorescence intensity at each region of interest. The RPR and native perfusion ratio were obtained along the length of the neo-oesophagus (Figure 1A) and at the oesophageal stump (Figure 1B), respectively. This was calculated by the software as the ratio of fluorescence intensity at each region of interest to that of the POR. The area with the highest perfusion at the gastric conduit was selected for anastomosis.

### 2.4. Postoperative Follow-Up

The patients were kept nil-per-oral postoperatively with a nasogastric tube on free drainage. Routine postoperative CT oesophagograms were performed 3–5 days after surgery to assess anastomotic integrity before initiating oral intake. Radiological anastomotic leak was defined as contrast extravasation at the oesophagogastric anastomosis on oesophagograms without associated clinical signs. Clinical anastomotic leak was classified as a deviation in clinical condition or treatment due to a proven or suspected leak.

Perioperative complications were classified using the Clavien–Dindo Classification [26] of surgical complications (Grade I: no intervention; Grade II: medical management; Grade III: invasive intervention, such as radiological procedures; and Grade IV: life-threatening complications warranting urgent return to theatre or ICU admission).

After discharge, follow-up was conducted by the surgeon and medical oncologist to review progress, tumour markers, and CT scans (chest, abdomen, and pelvis), when appropriate.

### 2.5. Outcomes and Statistical Analysis

The primary endpoints were the incidence of anastomotic leak (radiological or clinical). Secondary endpoints included major complications (medical or surgical) and 90-day mortality.

Clinical data were extracted for all selected patients. Statistical analyses were conducted using SPSS for Windows (Version 24, IBM Corporation, New York, NY, USA). Patient characteristics were analysed using frequency and descriptive statistics.

## 3. Results

This study included six patients (three males and three females) undergoing oesophagectomy, with a median age of 69 years (range: 61–79). Histopathological analysis revealed adenocarcinoma in half (3/6) of the cases, predominantly junctional tumours (Siewert II: 4/6). Most patients were non-smokers (5/6) and non-diabetics (5/6). Comorbidities included hypertension, hyperlipidaemia, and obesity (Table 1). Neoadjuvant therapy was based on the Chemotherapy for Oesophageal Cancer followed by Surgery Study (CROSS) protocol in five cases, with one patient receiving fluorouracil, leucovorin, oxaliplatin, and docetaxel (FLOT) chemotherapy. Surgical approaches comprised predominantly Ivor Lewis oesophagectomy (5/6), of which four utilised a hybrid technique, and one McKeown procedure.

Intraoperative ICG angiography confirmed robust perfusion in all cases, with native perfusion ratios exceeding 80% at the oesophageal stump. Anastomotic sites were selectively chosen based on maximal RPR, with ratios as follows: 95–100% (n = 2), 85–90% (n = 2), 84–87% (n = 1), and 80–85% (n = 1). Postoperatively, no anastomotic leaks were observed clinically or radiologically. Complications included three Clavien–Dindo grade II and two grade IV morbidities, all secondary to respiratory complications requiring antibiotics or reintubation (Table 2). The 90-day mortality rate was 0%.

## 4. Discussion

This preliminary study attempted to assess the feasibility of ICG fluorescence angiography using the SPY-PHI QP system’s built-in software for quantifying relative perfusion of the gastric conduit prior to surgical anastomosis construction. Objective quantification of tissue perfusion through this approach holds significant potential to improve surgical outcomes by mitigating complications associated with ischaemia, such as anastomotic leaks and conduit necrosis. By establishing the first portion of the duodenum as the POR, surgeons can select the anastomotic site along the neo-oesophagus based on the maximum RPR generated by the software. Our findings indicate that an RPR threshold exceeding 80% is safe for performing oesophagogastric anastomosis. Notably, no anastomotic leaks were observed in our cohort, and the 90-day mortality rate was zero, underscoring the clinical relevance of this perfusion-guided strategy.

The use of ICG traces its origins to 1955, when it was first developed by Kodak as an agent for photographic applications [27]. Later approved by the U.S. Food and Drug Administration (FDA) for medical use, ICG gained prominence in clinical settings for cardiac output measurement, hepatic function assessment, and ophthalmic angiography [28]. Over time, advancements have expanded its intraoperative utility across diverse surgical specialties, including breast, plastic, reconstructive, cardiovascular, and colorectal surgery [29].

Historically, surgeons have relied on subjective assessments such as gastric serosal colour, arterial pulsation, and bleeding at the conduit tip to evaluate perfusion [30,31]. However, these methods are often unreliable, especially in cases where ischaemic areas are not easily distinguishable [31]. For example, intestinal cyanosis caused by transient venous congestion may resolve without permanent damage, while bowel with early arterial occlusion—a more critical ischemic event—can initially appear normal [30]. Such limitations underscore the inherent challenges in relying on macroscopic cues alone. In this context, ICG’s near-infrared fluorescence emission and rapid metabolic clearance have positioned it as a transformative tool. Its ability to demonstrate instantaneous perfusion of surgical anastomoses can enhance procedural precision and optimise patient outcomes [32,33,34]. Supporting this, a meta-analysis of 11 studies reported a significantly reduced risk of postoperative anastomotic leak in patients undergoing ICG-guided oesophagogastric anastomosis compared to non-ICG approaches [19].

While ICG fluorescence imaging offers valuable dynamic perfusion assessment, its limitations warrant consideration. Studies have reported interobserver variability in interpreting fluorescence angiograms during gastric conduit perfusion evaluation in esophagectomy, though this inconsistency diminishes as surgeons gain experience and overcome the learning curve [35,36]. A key contributing factor is the phenomenon of ICG “creep,” where distinct bowel segments reach peak fluorescence intensity progressively over time, leading to potential misinterpretation [37]. To address this, quantification software tools based on fluorescence intensity have emerged as a solution, standardising assessments and reducing reliance on surgeons’ subjective interpretation.

The use of ICG fluorescence angiography to assess tissue perfusion has evolved through multiple software iterations and quantification methodologies, as documented in prior studies [34,36,38,39]. A study by Campbell et al. demonstrated a reduction in anastomotic leak rates from 20% to 0% in esophagectomy patients after adopting the SPY Elite System (LifeCell, Bridgewater, NJ, USA), using a perfusion threshold of 75% relative intensity compared to the gastric antrum [31]. In contrast, our study employed the SPY-PHI QP^®^ system (Stryker AB, Malmö, Sweden), an advanced iteration of fluorescence imaging technology with enhanced quantitative perfusion analysis [40]. While this system has been described in breast [23] and plastic surgery [41]—where a 25% ICG ratio threshold for flap-to-normal tissue perfusion reduced flap-related complications—its application to gastric conduit perfusion assessment during esophagectomy remains limited.

More recently, a prospective study involving 100 patients undergoing subtotal oesophagectomy with gastric conduit reconstruction reported a thirteen-fold increase in anastomotic leak when fluorescence intensity at the anastomotic site was ≤90% using real-time SPY-PHI/SPY-QP imaging [42]. In our cohort, application of an RPR threshold exceeding 80% was associated with the absence of anastomotic leak. This discrepancy in threshold values may be attributable to differences in reference point selection. In our study, the first part of the duodenum was used as the point of reference, based on the assumption that it represents maximal perfusion following division of the left gastric and short gastric vessels, thereby providing a standardised comparison. In contrast, Kajiyama et al. defined the reference point at the central anterior wall of the gastric conduit at the entry of the first right gastroepiploic branch [42].

This study has several limitations, and the results should be interpreted with caution. First, the small sample size (n = 6) limits statistical power and generalisability. Second, the single-centre, single-surgeon study design introduces potential selection bias. Additionally, the time interval between ICG administration and fluorescence analysis was not recorded, which may affect interpretation due to ICG’s gradual diffusion into ischemic tissue [37]. The lack of a control group precludes direct comparison with non-ICG techniques. We also acknowledge that although the SPY-PHI QP^®^ quantitative module has been reported in prior studies [23,41], our institution has not undertaken a formal independent validation. This pilot series reflects our initial implementation experience with the software. Finally, while the 80% RPR threshold aligned with favourable outcomes, variability in software algorithms across systems complicates universal threshold standardisation. In the future, larger, multicentred trials are required to validate these findings and refine perfusion quantification protocols.

## 5. Conclusions

This retrospective study demonstrates the adoption of quantitative ICG angiography using the SPY-PHI QP^®^ system to guide anastomotic site selection in oesophagectomy. These results suggested that objective perfusion quantification may enhance surgical decision-making and reduce anastomotic leak-related morbidity. While these early findings are encouraging, they should be interpreted cautiously given the limited sample size and non-comparative design.

## Figures and Tables

**Table 1:** Characteristics of patients undergoing ICG-guided oesophagectomy.

Case	Age	Gender	Siewart Classification	Histology	Smoker	Type 2 Diabetes	Other Comorbidities	Staging	Neoadjuvant Therapy
1	70	M	I	SCC	No	No	HTN	ycT3N0	CROSS
2	68	M	II	Adeno	Yes	Yes	Hyperlipidaemia	ycT2N0	CROSS
3	73	F	I	SCC	No	No	HTN, AVR	ycT2N0	CROSS
4	62	M	II	SCC	No	No	HTN	ycT3N2	CROSS
5	61	F	II	Adeno	No	No	Depression	ycT3N1	FLOT
6	79	F	II	Adeno	No	No	GORD, OSA, pulmonary hypertension, obesity	ycT3N0	CROSS (Capecitabine only)

SCC: squamous cell carcinoma; HTN: hypertension; Adeno: adenocarcinoma; AVR: aortic valve replacement.

**Table 2:** Details of surgery, gastric conduit perfusion, and postoperative outcomes.

Case	Surgery	Approach	Native Perfusion Ratio	Relative Perfusion Ratio	Anastomotic Leak		Complications	90-Day Mortality
					Radiological	Clinical		
1	Tri-incisional Oesophagectomy (McKeown)	Open	85–90%	80–85%	No	No	Grade IV: pneumonia requiring prolonged intubation and tracheostomy	No
2	Ivor Lewis Oesophagectomy	Hybrid	85–91%	85–90%	No	No	Nil	No
3	Ivor Lewis oesophagectomy	Hybrid	85–88%	95–100%	No	No	Grade IV: pneumonia/mucous plugging requiring reintubation	No
4	Ivor Lewis Oesophagectomy	Hybrid	85–90%	95–100%	No	No	Grade II: pneumonia with antibiotic treatment	No
5	Ivor Lewis Oesophagectomy	Hybrid	87–90%	85–90%	No	No	Grade II: pneumonia with antibiotic treatment	No
6	Ivor Lewis Oesophagectomy	Open	84–88%	84–87%	No	No	Grade II: pneumonia with antibiotic treatment	No

AL

Anastomotic leak

ICG

Indocyanine green

RPR

Relative perfusion ratio

NIR

Near-infrared

POR

Point of reference

ICU

Intensive care unit

FDA

Food and drug administration

CROSS

Chemotherapy for Oesophageal Cancer followed by Surgery Study

FLOT

Fluorouracil, leucovorin, oxaliplatin, and docetaxel

## Data Availability

The raw data supporting the conclusions of this article will be made available by the authors on request.

## Abbreviations

The following abbreviations are used in this manuscript.

AL	Anastomotic leak
ICG	Indocyanine green
RPR	Relative perfusion ratio
NIR	Near-infrared
POR	Point of reference
ICU	Intensive care unit
FDA	Food and drug administration
CROSS	Chemotherapy for Oesophageal Cancer followed by Surgery Study
FLOT	Fluorouracil, leucovorin, oxaliplatin, and docetaxel
